# Severe atypical iliac wing fracture associated with long-term bisphosphonate use

**DOI:** 10.1007/s00256-024-04738-9

**Published:** 2024-07-20

**Authors:** John Kelliher, George Rahmani, John J. Carey, Diane Bergin

**Affiliations:** 1 University Hospital Galway, Galway, Ireland; 2 University of Galway, Galway, Ireland

**Keywords:** Bisphosphonates, Pelvic fractures, Atypical fractures, Postmenopausal

## Abstract

**Background:**

Bisphosphonate use is associated with atypical non-traumatic fractures, which are most commonly seen in the femur.

**Case presentation:**

We report a 63-year-old postmenopausal woman who presented acutely with progressively worsening lumbar pain radiating to her left hip for 10 days. There was no antecedent trauma. On examination, the patient could not bear weight on her left leg due to the severity of the pain. Radiography and computed tomography of the pelvis demonstrated an iliac wing fracture which was treated conservatively. The patient had a significant past medical history of breast cancer and intense bisphosphonate use for several years which was discontinued 3 years previously. No discrete bone lesion was seen at the fracture site on computed tomography, and there was no evidence of metastatic disease elsewhere. A dual-energy X-ray absorptiometry scan showed the lowest bone mineral density *T*-score of − 1.2. A diagnosis of an atypical fracture related to long-term bisphosphonate therapy was made.

**Conclusion:**

To the best of our knowledge, this is the first reported case of an isolated iliac wing fracture associated with long-term bisphosphonate therapy in the literature. Whilst the incidence of such fractures is exceedingly rare, it is an important differential in patients with atypical fractures on long-term bisphosphonates.

## Introduction

Bisphosphonates inhibit osteoclast-mediated resorption and remodelling of bone which increases bone mineral density (BMD) and improves the bone microstructure [1]. As a result, bisphosphonates are used in osteoporosis and breast cancer treatment-induced bone loss. Whilst bisphosphonates have a relatively small side-effect profile, prolonged use leads to oversuppression of bone turnover and disruption of normal bone remodelling, causing the accumulation of microdamage which increases the risk of atypical fractures [2].

The incidence of atypical fractures linked to bisphosphonate use varies depending on factors such as treatment duration and patient demographics [3, 4]. They typically occur in older, postmenopausal women [5]. Fractures commonly associated with bisphosphonate use are atypical femoral fractures [3]. The incidence of atypical femoral fractures for female patients on continuous bisphosphonate treatment is 1/1000 per year, compared to 0.02/1000 for untreated women [5]. Pelvic fractures have also been reported but are much rarer [6–11]. Risk factors for atypical fractures include a longer duration of bisphosphonate use, which rapidly decreases after discontinuation, and Asian race [2]. Other risk factors include femoral bowing and glucocorticoid use [12].

Rates of bone loss associated with aromatase inhibitors, used to treat breast cancer, are reported to be at least twofold higher than healthy, age-matched postmenopausal women, with a significantly higher incidence of fractures [13]. As a result, oncology patients require higher cumulative doses of bisphosphonates compared to osteoporosis patients, which may also increase the risk of atypical femoral fractures [12]. In the AZURE trial, which is the largest trial to assess the adjuvant use of bisphosphonates in breast cancer patients, high-dose zoledronic acid 4 mg was administered every 3 to 4 weeks for 6 doses and then every 3 to 6 months to complete 5 years of treatment [14], a dose much higher than used in osteoporosis.

Atypical fractures associated with bisphosphonates commonly present with insidious onset of hip or groin pain and may lack a history of significant trauma. Fractures are typically found in the femur, distal to the lesser trochanter but proximal to the supracondylar flare, and are predominantly transverse or slightly oblique [4]. They can be bilateral at the time of presentation and are often associated with thickening of the lateral cortex and periosteal stress reaction, suggesting these are insufficiency fractures [4].

Here we describe the case of a 63-year-old woman diagnosed with an atypical iliac wing fracture after 10 years of bisphosphonate use.

## Case report

A 63-year-old postmenopausal female presented to the emergency department with acute, progressive severe lumbar pain radiating to her left hip for 10 days which began when she sat in a chair. On examination, the patient could not bear weight on her left leg due to the severity of the pain. There was no history of trauma or precipitating event. Blood tests were within normal parameters, including vitamin D, calcium, and parathyroid hormone levels. Her most recent dual-energy X-ray absorptiometry scan (G.E. Lunar Prodigy, Software version 17.0, NHANES III white female reference population) showed the patient’s lowest bone mineral density *T*-score to be 0.1.

The patient’s past medical history was significant for breast cancer diagnosed 14 years previously. She underwent a left breast-wide local excision and left axillary clearance. Following surgery, she had adjuvant chemotherapy, radiotherapy, and hormonal therapy with letrozole, an aromatase inhibitor, for 10 years. She was prescribed adjuvant therapy with high dose zoledronic acid 4 mg every 4 weeks for 6 months, followed by 4 mg every 3 and then 6 months for a total of 10 years. This had been discontinued 3 years previously. Other medical history included hypertension, for which she took losartan and escitalopram for depression and anxiety. She was also taking daily calcium and vitamin D supplements. There was no relevant family history of fractures, osteoporosis, or other bone disease.

**Fig. 1:**
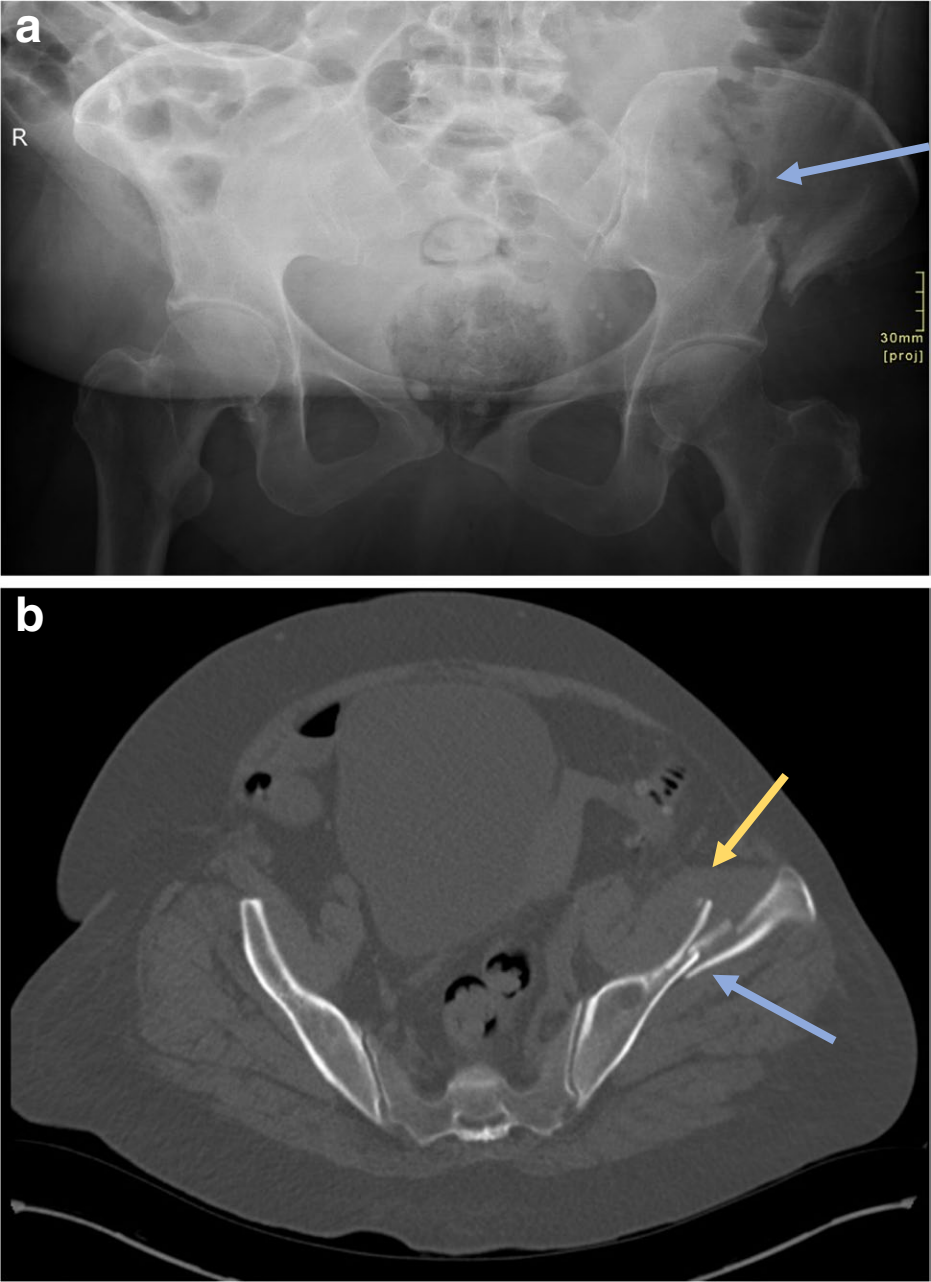
A left iliac wing fracture associated with bisphosphonate use in a 63-year-old woman presenting with lumbar pain radiating to her hip. **a** Anteroposterior pelvic radiograph showing a vertically orientated, displaced fracture of the left iliac wing (blue arrow). **b** Axial computed tomography of the pelvis without intravenous contrast showing a left iliac wing fracture (blue arrow) and soft tissue swelling in the overlying left iliacus muscle, likely haematoma (yellow arrow), without appreciable cortical thickening

Radiography revealed a vertically orientated, displaced fracture of the iliac wing (Fig. 1a). Computed tomography visualised the same fracture with a haematoma in the overlying left iliacus muscle (Fig. 1b). There was no discrete bone lesion at the site of the fracture on computed tomography, and there was no evidence of metastatic disease elsewhere on computed tomography thorax, abdomen, and pelvis to suggest a pathological fracture. The case was discussed with the national centre for the treatment of pelvic and acetabular fractures. At the time of review, the available imaging indicated progressive healing. Due to the location and nature of the fracture, the patient was treated conservatively, which involved orthopaedic input, pain management, fracture liaison service, and weight bearing as tolerated with physiotherapy input.

**Fig. 2:**
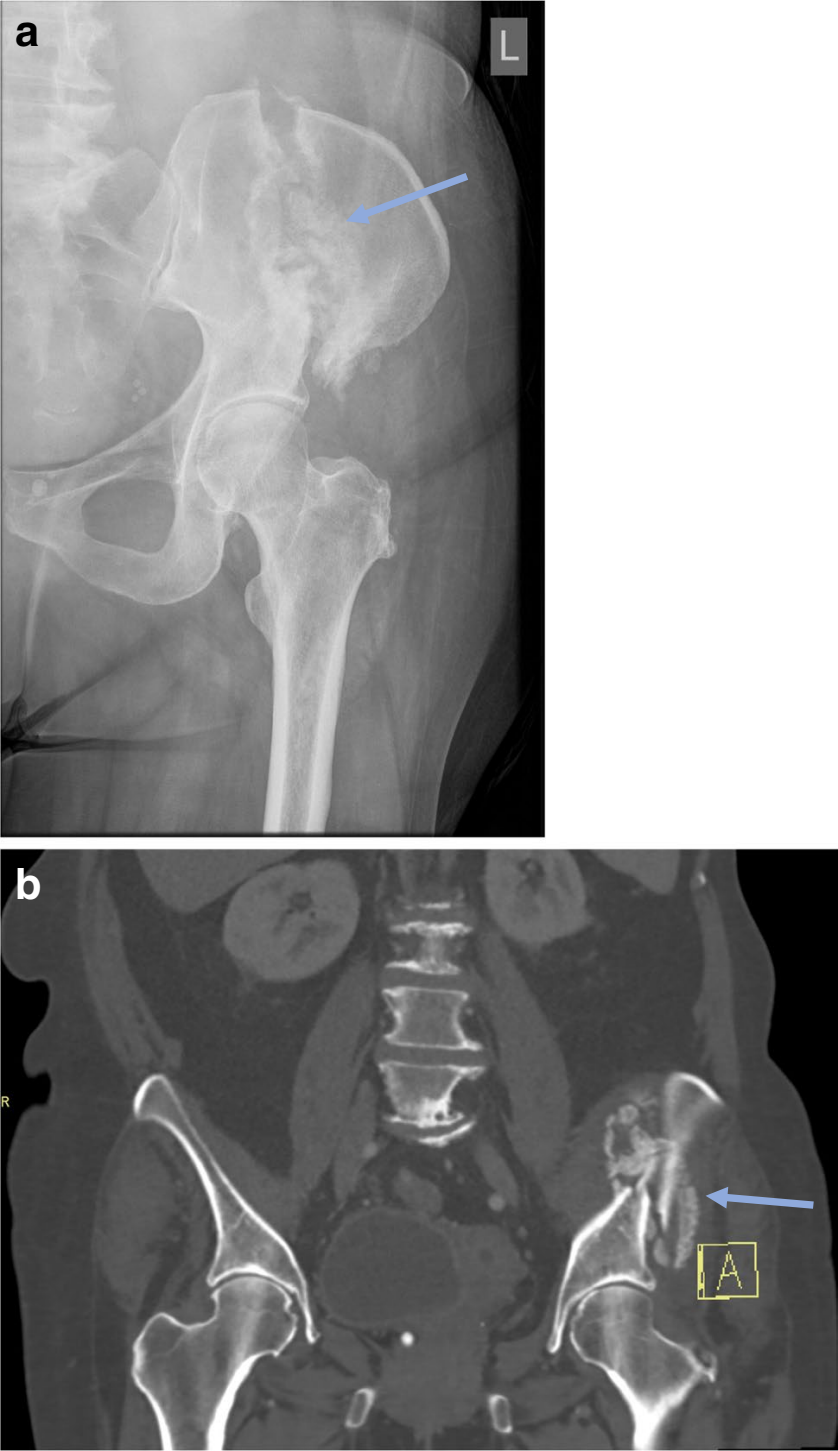
A left iliac wing fracture (blue arrow) associated with bisphosphonate use in a 63-year-old after 2 months. **A**nteroposterior pelvic radiograph showing florid callous formation at the fracture site (**a**) and corresponding coronal computed tomography of the abdomen and pelvis showing external callous formation (**b**)

**Fig. 3:**
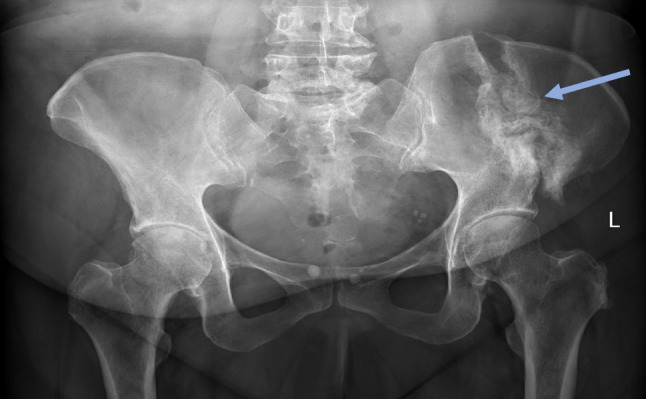
A left iliac wing fracture associated with bisphosphonate use with florid callous formation (blue arrow) after 4 months in a 63-year-old woman. Anteroposterior pelvic radiograph showing florid callous formation at the fracture site (blue arrow)

The patient’s pain improved during her hospital admission, and she began to mobilise with crutches. After 10 days, the patient was discharged and prescribed a course of teriparatide to promote fracture healing. She subsequently had a repeat dual-energy X-ray absorptiometry scan 2 months later which showed osteopaenia, with the patient’s lowest *T*-score being − 1.2 at the lumbar spine. Radiography after 2 months showed florid callus formation at the fracture site (Fig. 2a). Computed tomography after 2 months showed external callus formation (Fig. 2b). Radiography after 4 months also showed florid callous formation (Fig. 3). The patient was reviewed in outpatient clinics several times and finally at 17 weeks could walk again independently.

This case report describes an isolated acute iliac wing fracture in a 63-year-old woman with no history of trauma and normal bone mineral density. As there were no other predisposing factors, a diagnosis of an atypical fracture related to bisphosphonate therapy was made. Conservative treatment with physiotherapy, analgesia, and teriparatide has resulted in progressive healing of the fracture.

## Discussion

Long-term bisphosphonate use is associated with atypical femoral fractures [2] and is a rare cause of hip and pelvic fractures, with the first reported cases in 2005 [6]. To the best of our knowledge, this is the second reported case of an ilium fracture due to long-term bisphosphonate use and the first isolated iliac wing fracture. Eight cases of pelvic fractures due to bisphosphonates have been reported in the literature, and only one involving the ilium [6–11]. The other pelvic fracture sites included the sacrum, pubic rami, and ischium. All the patients were females presenting with atraumatic or low-energy trauma fractures. Seven were postmenopausal, which is a common occurrence in bisphosphonate-associated fractures [5]. The primary indication for bisphosphonate therapy was osteoporosis and osteopaenia, with one patient having concomitant breast cancer. The duration of bisphosphonate use varied, ranging from 3 to 16 years, with longer duration of use being a known risk factor [2]. Four patients had a history of oestrogen or hormone replacement therapy (HRT). The combination of another antiresorptive agent may have led to further suppression of bone turnover [15]. One patient was Japanese, with Asian race also being a risk factor [2]. Other risk factors include femoral bowing and glucocorticoid use [12]. These findings highlight the complexity of bisphosphonate-associated fractures in postmenopausal women, warranting careful consideration of individual risk factors and treatment duration.

A task force formed by the American Society for Bone and Mineral Research to address the association between long-term bisphosphonate use and atypical fractures in patients with osteoporosis concluded that the benefits of bisphosphates in preventing fractures, in particular vertebral and hip fractures, significantly outweigh the relatively small absolute risk of atypical fractures attributed to chronic use [4].

The patient’s symptoms of progressively worsening pain over 10 days were in keeping with the prodromal symptoms of dull or aching pain often described with atypical fractures. A retrospective assessment of previous imaging to look for classical findings associated with long-term bisphosphonate therapy, such as cortical thickening, is advised. Conventionally radiographs are the first-line imaging modality, followed by magnetic resonance imaging and nuclear scintigraphy if clinically required. It has been suggested the need to screen for atypical fractures using dual-energy X-ray absorptiometry scans with full femur-length images in high-risk patients, such as those on long-term antiresorptive drugs like bisphosphonates or denosumab [16]. However, such testing would not have been helpful in this case as the pelvis is not seen on routine dual-energy X-ray absorptiometry scans. Furthermore, there is no criteria for the early detection of atypical pelvic fractures associated with bisphosphonates, as these fractures have never been reported [17]. The patient was started on a 2-year trial of teriparatide, a synthetic parathyroid hormone. From the low-quality of evidence available, the American Society for Bone and Mineral Research task force recommends considering teriparatide for patients with atypical fractures associated with bisphosphonate use [4]. Interestingly, our patient had completed her treatment with zoledronic acid 3 years previously, but the skeletal effects of zoledronic acid may persist for years following treatment [18].

In summary, this case report discusses an unusual presentation of an atypical iliac wing fracture associated with bisphosphonate use. To the best of our knowledge, it is the first reported isolated iliac wing fracture associated with long-term bisphosphonate therapy in the literature. Whilst the incidence of such fractures is very low, it is perhaps an important differential in patients presenting with atypical fractures even after cessation of long-term bisphosphonates.

## Data Availability

All data generated or analysed during this study are included in this published article (and its supplementary informational files).
